# Molecular cytogenetic analysis of early spontaneous abortions conceived from varying assisted reproductive technology procedures

**DOI:** 10.1186/s13039-016-0284-2

**Published:** 2016-10-12

**Authors:** Tonghua Wu, Biao Yin, Yuanchang Zhu, Guangui Li, Lijun Ye, Chunmei Chen, Yong Zeng, Desheng Liang

**Affiliations:** 1 Shenzhen Key Laboratory for Reproductive Immunology of Preimplantation, Shenzhen Zhongshan Institute for Reproduction and Genetics, Fertility Center, Shenzhen Zhongshan Urology Hospital, No. 1001 Fuqiang Road, Futian District, Shenzhen, 518045 China; 2 The State Key Laboratory of Medical Genetics of China, Central South University, No. 110 Xiangya Road, Kaifu District, Changsha, 410013 China

**Keywords:** Spontaneous abortion, Aneuploidy, ART, Maternal age, FET

## Abstract

**Background:**

Spontaneous abortion (SA) is the most common complication of pregnancy, and chromosome aberrations are the principal cause of the first trimester abortuses in natural conception (NC) The increasing use of assisted reproductive technology (ART) has raised concern about chromosome abnormalities in ART-initiated pregnancies. Up to date, the literature on the risk of aneuploidy in failed pregnancies among various ART factors remain limited and inconclusive. This study aimed to explore the genetic etiology of pregnancy loss conceived from varying ART procedures.

**Results:**

A total of 560 cases of villus that were successfully collected and performed molecular karyotype analysis were enrolled in present research, including 92 cases of NC, 340 cases of in-vitro fertilization (IVF) and 128 cases of intracytoplasmic sperm injection (ICSI). There was no statistical difference in the distribution of karyotyping results and the aneuploidy rate of each individual chromosome among NC, IVF and ICSI group. Both the total chromosomal abnormality rate and the one chromosome aneuploidy rate were increased with maternal age. Compared with fresh ET abortion group, frozen-thawed embryo transfer (FET) abortion group had elder maternal age (34.68 ± 4.73 years vs. 33.41 ± 4.48 years, *P* = 0.003) but lower chromosomal aberration rate of abortus (58.33 % vs. 67.50 %, *P* = 0.040). A slightly higher incidence of chromosome segmental abnormalities was found in FET than in fresh ET abortion (5.26 % vs. 2.08 %, *P* = 0.066).

**Conclusions:**

Chromosomal abnormality of fetus is the main cause of SA in the first trimester, no matter pregnancies are conceived through NC, IVF or ICSI. ART is a relatively safety treatment, and it does not enhance aneuploidy rate of abortus. The FET is bad for ongonging pregnancy and the aneuploidy rate were increased with maternal age.

## Background

The most frequent pregnancy related complication is spontaneous abortion (SA). Approximately 10–15 % of all clinical pregnancies end in SA, of which a large proportion involves genetically abnormal fetuses. The earlier pregnancy loss occurs, the greater likelihood of genetics causes [1]. Multiple cytogenetic studies have demonstrated that the rate of chromosome aberrations in the first trimester abortuses range from 50 to 85 % in natural conception (NC) [2]. Chromosomal anomaly spectrum in early pregnancy loss consists of numerical abnormalities (86 %), mosaicisms (8 %) and structural abnormalities (6 %) [3].

Cytogenetic karyotype analysis of villus, which used to be considered as a standard method to detect fetal chromosome abnormalities, is not only labor intensive but also subject to a significant failure rate of cell culture. Considerable efforts have been put into developing molecular approaches that achieve to diagnose aneuploidies rapidly and precisely without depending on cell culture. Multiplex ligation-dependent probe amplification (MLPA), a method for relative quantification of more than 40 different DNA sequences simultaneously in one rapid and economical assay requiring only 20 ng of DNA [4], enables the detection of aneuploidy and unbalanced chromosomal rearrangements in products of conception (POC) [5]. It has been validate to facilitate routine examination of chromosome abnormalities in SA using MLPA combine with an fluorescence *in situ* hybridization (FISH) or quantitative fluorescent- polymerase chain reaction (QF-PCR) for mosaicisms and polyploidy [6, 7].

The increasing use of assisted reproductive technology (ART) has generated concern about chromosome abnormalities in ART-initiated pregnancies on account of the higher miscarriage rates that have been reported [8–10]. Some factors might contributing to the higher incidence of SA in sterility population include the use of ovulation stimulants, advanced maternal age (AMA), intracytoplasmic sperm injection (ICSI), fertilization and culture in vitro, cryopreservation of embryos or gametes, and so forth [11]. The ART procedures are completely different from natural pregnancy; it would potentially indicate an increased frequency of aneuploidy in the first trimester pregnancy accounting for bypassing the natural mechanisms of fertility. However, up to date, the literature on the risk of aneuploidy in SA among various ART factors remains limited and inconclusive [11–13]. In order to explore the genetic etiology of pregnancy loss conceived from varying ART procedures without a bias causing by cytogenetic karyotype, MLPA, combined with FISH and QF-PCR were applied to molecular karyotype analysis of early fetal demise in ART.

## Methods

### Population

From March 2011 to July 2015, all cases of singleton pregnancies that ended in SA in the first trimester and agreed to analyze chromosome of abortuses after performing dilation and curettage (D&C) at Shenzhen Zhongshan Urology Hospital were included in the present study. These cases of SA were conceived by conventional in-vitro fertilization (IVF), ICSI and NC. The study was approved by the Research Ethics Committee of Shenzhen Zhongshan Urology Hospital and informed consent was obtained from each patient prior to the study.

### ART procedure

ART patients received ovarian stimulation using Gonadotrophine-releasing hormone agonist and Gonadotrophine. After meeting the ultrasonographic criteria for follicular maturity, a single dose of human chorionic gonadotropin (hCG) was administered. Transvaginal follicular aspiration was performed 36 h after hCG administration. Oocytes were treated by conventional IVF or ICSI 4 h after ovum pick-up. Embryos were transferred 3–5 days after the follicular aspiration, or frozen embryos were thawed and then were transferred in frozen-thawed cycles.

### Sample collection

Villi or fetal parts were dissected free from any maternal decidua or blood clots under the dissecting microscope and repeatedly washed in normal saline to avoid maternal cell contamination (MCC). The samples were divided into two parts; one is for DNA examination and another is for FISH analysis if necessary.

### DNA extraction

Genomic DNA of tissue and maternal blood were extracted using QIAamp DNA/Blood Mini Kit (Qiagen, Germany) according to the manufacturer’s instructions. The exacted DNA was quantified by spectrophotometer (Shimadzu, Japan), and then stored at -20 °C.

### QF-PCR for sample quality control

In order to eliminate the samples containing MCC [14], amplifications of microsatellite markers were carried out with all DNA villi samples and maternal blood. QF-PCR products were separated on ABI 3500 Genetic Analyser and analyzed with the GeneMapper 4.0 software package (Applied Biosystems, USA).

### MLPA for aneuploidy analysis

DNA samples were tested by MLPA with SALSA P036, P070 and P181 probe mixes (MRC-Holland, The Netherlands) following the manufacturer’s protocols. Copy number variation at both two subtelomeres and centromere of any individual chromosome indicated a whole chromosome aneuploidy. Increased or decreased dosage at one subtelomere or/and centromere of one chromosome indicated a segmental aneuploidy.

### FISH for polyploidy and mosaicisms analysis

If MLPA result excluded any aneuploidy, the slide was processed interphase FISH with three different color probes to three different chromosomes. Chromosomes’ FISH probes were commercial products (AneuVysion Assay Kit) available from Vysis (Abbott Molecular, USA). Calculation and analysis of hybridization signals for each probe were conducted using a fluorescent microscope (Nikon, Japan).

### Statistical analysis

Statistical analysis was performed with IBM SPSS Statistics 21 software. Mean ± standard deviation was compared by using the independent sample *t* test and ANOVA for continuous variables and proportions were compared with the chi-squared test for categorical variables. Differences were considered significant if *P* < 0.05.

## Results

A total of 560 cases of villus were successfully collected in the research, all the samples were not excluded from the study due to MCC. The mean age of female spouse did not show significant difference among three groups (NC *n* = 92, IVF *n* = 340 and ICSI *n* = 128). According to diagnosis results, the molecular karyotyping results were cataloged as euploidy, one chromosome aneuploidy, one chromosome segmental abnormality, multiple chromosme abnormality, polyploidy and uniparental disomy (UPD). Among in NC, IVF and ICSI group, distribution of karyotyping results was similar (*P* > 0.05) (Table 1).

**Table 1:** Distribution of molecular karyotyping results

	NC group	IVF group	ICSI group	*P*_*a*_ value	Fresh ET group	FET group	*P*_*b*_ value
Euploidy	38 (41.30 %)	127 (37.35 %)	46 (35.94 %)	0.706	78 (32.50 %)	95 (41.67 %)	0.040*
One chromosome aneuploidy	35 (38.04 %)	164 (48.24 %)	61 (47.66 %)	0.210	135 (56.25 %)	92 (40.35 %)	0.001*
One chromosome segmental abnormality	5 (5.43 %)	13 (3.82 %)	5 (3.91 %)	0.781	5 (2.08 %)	12 (5.26 %)	0.066
Multiple chromosme abnormality	11 (11.96 %)	35 (10.29 %)	15 (11.72 %)	0.851	22 (9.17 %)	27 (11.84 %)	0.345
Polyploidy	3 (3.26 %)	1 (0.29 %)	0 (0.00 %)	--	0 (0.00 %)	1 (0.44 %)	--
Uniparental disomy (UPD)	0 (0.00 %)	0 (0.00 %)	1 (0.78 %)	--	0 (0.00 %)	1 (0.44 %)	--
Total	92	340	128	--	240	228	--

Values are number of patients and (percent) with given molecular karyotyping results

*P*_*a*_ value: NC group vs. IVF group vs. ICSI group

*P*_*b*_ value: fresh ET group vs. FET group

*: *P* < 0.05

Of all the ART abortuses, 240 cases were derived from fresh embryo transfer (ET) and 228 cases were derived from frozen-thawed embryo transfer (FET). Compared with FET, fresh ET abortions presented statistical younger maternal age (33.41 ± 4.48 years vs. 34.68 ± 4.73 years, *P* = 0.003) but significant higher chromosomal aberration rate (67.50 % vs. 58.33 %, *P* = 0.040). Molecular karyotyping distribution of these two groups was listed in Table 1.

**Fig. 1:**
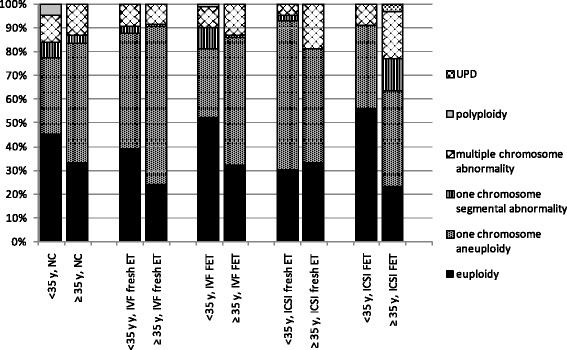
Stacked column diagram of molecular karyotyping results between < 35 years and ≥ 35 years subgroup

The groups were further subdivided by maternal age (<35 and ≥ 35 years), while IVF group and ICSI group were further subdivided into fresh ET group and FET group. Proportion of molecular karyotyping results among subgroups was shown in Fig. 1. Notable decreases of euploidy rate were observed with increasing maternal age in all pairs, which were listed in Fig. 1. In each subgroup, one chromosome aneuploidy was the most prevalent abnormality since it took up the proportion of aberrance from 52.17 % to 87.04 %. Incidence of multiple chromosome abnormality demonstrated a resemblance between < 35 years and ≥ 35 years subgroup in NC pair, IVF fresh ET pair and IVF FET pair, while a rise by comparing elder subgroup to younger subgroup in ICSI pair, ICSI fresh ET pair and ICSI FET pair.

**Fig. 2:**
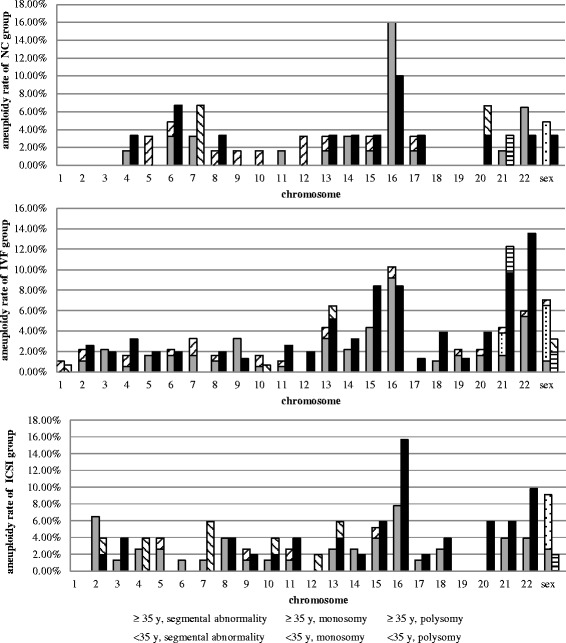
Aneuploidy rate of NC, IVF and ICSI group that subdivided by maternal age (< 35 years and ≥ 35 years) for each individual chromosome exhibiting monosomy, polysomy and segmental abnormality

Aneuploidy rate of three groups that subdivided by maternal age (< 35 and ≥ 35 years) for each individual chromosome exhibiting monosomy, polysomy and segmental abnormality were shown separately in Fig. 2. monosomy was only detected in chromosome 21 and sex chromosome. Chromosome 16 and 22 were two chromosomes most frequently involved in aneuploidy. Significant differences were found in chromosome 21 (4.32 % vs. 12.26 %, *P* = 0.007) and chromosome 22 (5.95 % vs. 13.55 %, *P* = 0.017) in IVF group. There was no statistical difference in aneuploidy rate of each individual chromosome in any of the maternal age categories among NC, IVF and ICSI group (*P* > 0.05). Patterns of aneuploidy rate for each individual chromosome varied by maternal age were similar among three groups except for chromosome 16 which showed a decline in NC group, a draw in IVF group and an increase in ICSI group with AMA.

## Discussion

Spontaneous abortion is the most frequently pregnancy related complication and more than half are caused by chromosomal abnormalities no matter who conceived through natural conceived or ART process. Some researchers have reported that ART-initiated pregnancies might account for higher miscarriage rates. Hitherto, the exactly incidence and distribution of chromosome aberrations remain limited and inconclusive. In this retrospective research we found that genetic defect of fetus is the main cause of SA in the first trimester, different conceived methods would not enhance aneuploidy rate of abortus, and the advanced maternal age is the only related factor with increasing aneuploidy.

Our data reveals that no matter pregnancies are conceived by NC, IVF or ICSI, chromosomal abnormalities account for the majority of first trimester losses with rates ranging from 59.70 to 64.06 % and no statistical differences in the distribution of karyotyping results were found. It consists with other studies which have shown ART did not increase cytogenetic risk compared to NC [12, 15]. Furthermore, the rate of euploidy was similar at the same maternal age subgroup among in NC, IVF and ICSI (< 35 years: 45.16 % vs.44.86 % vs.41.56 %, *P* > 0.05; ≥ 35 years: 33.33 % vs.28.39 % vs.27.45 %, *P* > 0.05), confirming previous studies in demonstrating no correlation in aneuploidy rates between conventional IVF and ICSI groups.

We further investigated on the variation patterns of aneuploidy rate for each individual chromosome by maternal age among NC, IVF and ICSI group, which is seldom reported in previous research. A few chromosomes have distinguishing features on the variation tendency or fluctuation range among different mode of conception, such as chromosome 16, 21 and 22. Present study is just an initial glance at this point, it should be noted that the limitation is small sample size of most abnormal chromosomes in each conception group. Therefore, further studies with larger aneuploid sample size will be needed.

Polyploidy, mainly arisen by dispermy for triploidy and failure of cytokinesis for tetraploidy, it is mostly lost during the first or second trimester [16], and the frequencies of triploidy and tetraploidy are 3 % ~ 8 % and 0 % ~ 6 %, respectively [7, 16, 17]. In present study, triploidy abortus were observed in 3.26 % of NC cases and 0.29 % of IVF cases, but not found in ICSI cases. They were slightly lower than other researches, probably relate to that FISH for polyploidy analysis was only performed to samples showing a normal MLPA result in our study. It leads to those polyploid plus aneuploid cases are categorized as aneuploidy [6].

In our present study, the aneuploidy rate was increased with maternal age in IVF and NC groups, which might be explained by several molecular mechanisms including errors in recombination, improper spindle formation and microtubult-kinetochore interations, defects in the spindle assembly checkpoint, reduction of cohesion, this is consistent with other researcher [18, 19]. Therefore, the society ought to encouage the female to give birth before 35 year in order to aviod the miscarriage.

FET is an important component of ART treatments since it could enhance the cumulative pregnancy rate, but after the cooling and thawing processes, the embryos or blastomeres might damage because their genetics apparatus are more vulnerable under extreme factors [20]. In order to investigate the genetic factor to SA in the first trimester of FET, we compared FET abortion group with fresh ET abortion group and our results demonstrated there was a significantly lower chromosomal aberration rate but with a statistically elder maternal age in FET abortion group. Considering that the early SA rate of FET cycles was significantly higher than that of fresh ET cycles in our center (17.55 % vs. 22.35 %, *P* = 0.000), we presume that the decreasing aneuploidy rate in FET is probably caused by the effect of cryopreservation on embryos’ developmental potential which leads some euploid embryo to stop the growth.

Comparing FET subgroup with fresh ET subgroup of identical maternal age and artificial insemination way, only ≥ 35 years ICSI pairs indicated a contrary variation tendency of euploidy rate to other pairs. This difference is probably due to a high incidence of one chromosome segmental abnormality in ≥ 35 years ICSI FET subgroup (13.33 %) but no case in fresh ET pair. We also observed a similar phenomenon about the prevalence of one chromosome segmental abnormality in < 35 years IVF FET and fresh ET pair (8.75 % vs. 2.86 %, *P* = 0.153). As a slightly higher incidence of chromosome segmental abnormalities was found in FET than in fresh ET abortion (5.26 % vs. 2.08 %, *P* = 0.066), we infer it might be a synergy of the injury induced by frozen-thawed and the growth retardation caused by subtelomeric imbalances. However, large sample size of chromosome segmental abnormalities is required for further validation.

## Conclusions

In summary, chromosomal abnormality of fetus is the main cause of SA in the first trimester, no matter pregnancies are conceived through NC, IVF or ICSI. IVF and ICSI do not enhance aneuploidy rate of abortus, which relatively affirm the safety of ART in a genetic perspective. Maternal age is positive correlated with aneuploidy rate and FET will increase SA.

## Abbreviations

AMA: Advanced maternal age
ANOVA: One way analysis of variance
ART: Assisted reproductive technology
D&C: Dilation and curettage
ET: Embryo transfer
FET: Frozen-thawed embryo transfer
FISH: Fluorescence *in situ* hybridization
hCG: Human chorionic gonadotropin
ICSI: Intracytoplasmic sperm injection
IVF: In-vitro fertilization
MCC: Maternal cell contamination
MLPA: Multiplex ligation-dependent probe amplification
NC: Natural conception
POC: Products of conception
QF-PCR: Quantitative fluorescent- polymerase chain reaction
SA: Spontaneous abortion
UPD: Uniparental disomy
